# The British Association and Anæsthetics

**Published:** 1908-09-19

**Authors:** 


The Hospital

A Journal of

The Medical Sciences and Hospital Administration.

New Series. No. 80, Vol. III. [No. 1152, Vol. XLIV.] SATURDAY, SEPTEMBER 19, 1908.

### THE BRITISH ASSOCIATION AND ANÆSTHETICS.

There has been an unusually large proportion of papers having strictly medical interest read before this year's meeting of the British Association for the Advancement of Science, and no topic of greater interest and importance has been under discussion than that to which the physiological section has devoted a large part of its attention—to wit, the evergreen chloroform problem. Perhaps the most important contribution to this Section was the paper read by Dr. Hewitt, in which that gentleman summed up the views which he has already publicly expressed at various times in the past few years, notably at the discussion held by the Medico-Legal Society earlier in the year. Dr. Hewitt's crusade is conducted with the object of improving the administration of general anæsthetics in this country, an object so laudable that it is at first sight a little surprising to find the profession as a whole a trifle lukewarm in his support. We are convinced that the reason of this is not based on any difference of opinion as to the worthiness of the object striven for, but purely from doubt whether his programme is that best calculated to achieve it.

Let us consider very shortly the elements of the question, and first those points upon which unanimity is complete, or at least greatest. The outstanding fact which has impressed so many people, laymen as well as medical practitioners, is the steady and considerable increase in late years of the number of deaths during anæsthesia reported to the coroners, more especially in London. There are two factors, other than a deteriorated standard of proficiency among administrators, which may have had some effect in the causation of this. One is the very large increase in the number of annual administrations, and the modern tendency to operate on cases which are practically *in extremis* in the hope, often realised, of snatching a brand here and there from the burning. The other is the much more complete notification of all these cases to the coroners now than formerly. The effect of these influences must be estimated before any default can be imputed to the medical profession in bulk as unskilful administrators. Let us at once record our conviction, not only that the standard of administration has been steadily raised year by year, thanks largely to the influence of Dr. Hewitt and other teachers of repute during the last decade or two, but also that it is certainly higher in these islands—and especially in London—than outside them.

Admitting at the same time that there is still room, and pressing need, for further improvement, the question arises how this is to be secured. Improved education is obviously the key to the problem, and any regulations or devices whatsoever which will tend in that direction must have the hearty approval of every thinking medical man. But Dr. Hewitt goes further: he would see the administration of anæsthetics in towns, and even in the country, restricted as far as possible to specialists who shall do nothing else but practise the administration of anæsthetics. He would see at every hospital a resident anæsthetist, whose duty it shall be to administer, or to supervise and be responsible for, every anæsthetic not given by the visiting anæsthetists. In other words, he would build up a new special department of medical practice as distinct from the duties of what we call general practice as are opsonic determinations or radical mastoid operations, for example. Now we have once before pointed out (The Hospital, June 8, 1907, p. 260) that the study and practical work necessary to turn out a first-class anæsthetist are trivial compared with those necessary for a gynæcologist, ophthalmic surgeon, etc., of equal proficiency; and, in fact, that any man of the mental calibre of the resident medical officers of the large hospitals in this country can, and, as a rule, does, so perfect himself in this work that it is ridiculous to attempt to restrict him from its subsequent performance in private practice. Moreover such restriction is antagonistic to the educational reforms suggested, and quite rightly suggested. To render it impossible for a man to gain admission to the Medical Register without a fair knowledge of the administration of general anæsthetics is an admirable scheme; but then practically to forbid him to attain real expert proficiency during his subsequent training as a house surgeon or house physician by subjecting him to the tutelage of a specialist, and even depriving him of his opportunities altogether, is to stultify the educational advantages already pressed upon him. That this view has commended itself to the junior members of the profession in

London is evident enough from the fact that the posts of resident anæsthetist established at several metropolitan hospitals lately, chiefly because of the enthusiastic advocacy of Dr. Hewitt, are proving extremely and increasingly difficult to fill. There is one such post being advertised at this moment, to which even a very handsome and increased emolument has failed to attract any candidate for the greater part of a year. The truth is that medical education is now so prolonged and so costly, and house appointments take up so much additional time, that a man who has filled them is no longer young, and if he is ever to make a living he must settle down without the delay occasioned by an additional appointment which will teach him nothing he has not already had ample opportunity for learning.

With Dr. Hewitt's educational policy we are in full accord. We agree with him in the four reforms he suggests, which are—to prevent unqualified persons from giving anæsthetics; to improve the anæsthetic departments in hospitals; to make a thorough course of instruction compulsory for every student; and to compel hospital authorities to register every administration within their institutions. We further agree with the anonymous anæsthetist who wrote a letter to the *Times* of September 9, advocating greater independence for and recognition of anæsthetists in medical schools and elsewhere; but we do not agree that it is either necessary, advisable, or even useful to make of anæsthetics a specialty, only to be practised by those who confine themselves to it.

